# Romantic partner undermining in a behavioral weight loss intervention

**DOI:** 10.1007/s10865-025-00597-4

**Published:** 2025-08-08

**Authors:** Marny M. Ehmann, Nicole T. Crane, Reena S. Chabria, Danielle Arigo, Meghan L. Butryn

**Affiliations:** 1 Department of Psychological and Brain Sciences and Center for Weight, Eating and Lifestyle Sciences (WELL Center), Drexel University, Philadelphia, PA USA; 2 Department of Psychology, Rowan University, 201 Mullica Hill Rd, Robinson Hall, Glassboro, NJ 08028 USA; 3 Center for Weight, Eating and Lifestyle Sciences (WELL Center), Drexel University, Philadelphia, PA USA

**Keywords:** Social influences, Social undermining, Obesity, Behavioral weight loss

## Abstract

Romantic partner undermining is a type of negative social influence that has the potential to make weight loss more challenging and consists of two components: (1) undermining behaviors, or overt romantic partner behaviors including criticism/complaint of weight control or interference behaviors (e.g., offering up high calorie foods), and (2) undermining attitudes, including negative beliefs that a romantic partner has about their significant other’s weight loss attempts. The present study was the first to the authors’ knowledge to investigate romantic partner undermining attitudes and behaviors reported by participants enrolled in in a behavioral weight loss (BWL) intervention, a first line treatment for weight loss. The study also examined whether partner undermining attitudes and behaviors differed by relationship quality and between those who did and did not choose their romantic partner to serve in a support role in the intervention. Participants (*N* = 128) enrolled in a group-based BWL intervention reported on perceived romantic partner undermining attitudes and behaviors at baseline and 3 months (end of the intensive period of BWL intervention). Results showed that participants perceived minimal romantic partner undermining at baseline (attitudes *M* = 2.2 ± 0.7, behaviors *M* = 2.3 ± 1.2) and 3 months (attitudes *M* = 2.0 ± 0.6, behaviors *M* = 1.9 ± 1.1). However, participants reported a significant reduction in total undermining attitudes (*M* change = − 0.2 points) and behaviors (*M* change = − 0.4 points) from baseline to 3 months (*p*s <.05), highlighting the potential of group-based BWL interventions to mitigate romantic partner undermining without explicit content focused on undermining. Future research should examine romantic partner undermining across time in varying weight control and relationship conditions and measure undermining at the daily or weekly level to reduce recall bias and improve ecological validity.

## Introduction

Close to 75% of U.S. adults have a BMI in the overweight or obese range (BMI ≥ 25 kg/m^2^; Fryar et al., 2020) and are at heightened risk for weight-related adverse health outcomes and poor quality of life (Avila et al., 2015; Kroes et al., 2016; Sarma et al., 2021; Saydah et al., 2014). Social cognitive theory posits that influences in one’s social network (e.g., family, friends) affect one's ability to modify lifestyle behaviors for weight loss (Bandura, 2001; Enriquez & Archila-Godinez, 2022). The influence of a romantic partner may be especially potent given the highly interdependent nature of the relationship and behaviors that co-exist in the same environment (Dailey, 2018).

A large body of research has shown that social support from a romantic partner facilitates weight loss by enhancing self-regulation, promoting autonomous motivation, providing validation and praise, inducing accountability, and/or lending tangible assistance (Dailey, 2019; Dailey et al., 2016, 2021; Gettens et al., 2018; Gorin et al., 2013, 2020; Karfopoulou et al., 2016; Powers et al., 2022; Simpson & Campbell, 2013; Wing & Jeffery, 1999). However, in addition to or instead of social support, the influences of romantic partners may also be negative in nature and discourage engagement in weight control behaviors (Ogden & Quirke-McFarlane, 2023). Compared to positive social support, negative social influences from romantic partners have been largely under-addressed in weight loss research despite some evidence that negative social influences may exert *greater* control over behavior (DeLongis et al., 2004; Duffy et al., 2002). Negative social interactions can induce perceptions of social threat, which can provoke intense behavioral and emotional reactions (Arigo et al., 2022; Chogahara, 1999).

Specifically, *social undermining* is a type of negative social influence that is distinct from both a lack of positive social support (i.e., the absence of support) and negative social control, or using negative interactions to facilitate health behaviors (e.g., harsh criticism for dietary lapses). Social undermining has the potential to make weight loss more challenging and consists of two primary components: undermining behaviors and undermining attitudes (undermining attitudes are sometimes referred to as “undermining motivations”; e.g., Dailey et al., 2022; Mackert et al., 2011). Undermining behaviors include overt partner behaviors that may hinder weight control efforts, including goal-interfering actions and criticism/complaints of weight control efforts. For example, a romantic partner might engage in an undermining behavior by offering up foods that are inconsistent with a reduced calorie diet, and the participant may abandon healthier eating habits to appease their partner (Ball et al., 2010; Cruwys et al., 2015). Qualitative and cross-sectional studies have found that undermining behaviors are a critical barrier to weight control (Dailey, 2018; Hardcastle & Hagger, 2011; Harp, 2013; Metzgar et al., 2015; Rogerson et al., 2016; Romo & Dailey, 2014; Theiss et al., 2016; Whale et al., 2014) and contribute to reduced confidence in controlling eating behavior, unhealthier eating habits, and weight gain (Alshehri et al., 2021; Henry et al., 2013; Rieger et al., 2018; Wang et al., 2014).

Undermining attitudes are negative attitudes that a partner has about their significant other’s weight loss attempts. Common undermining attitudes, synthesized by Dailey et al. (2022), include the participant’s perception that their romantic partner believes: (1) the participant should not lose weight, (2) their weight control behaviors are an imposition on them, (3) their weight control efforts are disruptive to the relationship status/quality, (4) their weight control behaviors are unhealthy, (5) their focus on weight control behaviors is a negative reflection of the partner’s weight status or behaviors, and (6) their weight control behaviors are excessively stressful. Aligned with the social cognitive theory, the beliefs of one’s romantic partner may serve as a form of feedback that can influence an individual’s thoughts and behaviors (Bandura, 2001; Schunk & DiBenedetto, 2020). For an individual attempting to lose weight, their perception of their partner’s attitudes about their weight loss pursuit (e.g., “My partner thinks my weight loss behaviors impose on their lifestyle”) may influence self-efficacy, intentions for behaviors, and ultimately engagement in weight control behaviors. Indeed, this link has been supported by research by Dailey et al. (2022), which found that the romantic partner beliefs that their significant other’s weight loss efforts are unhealthy or an imposition on the romantic partner were negatively related to weight management behaviors, whereas partner perception that weight loss was unnecessary was positively associated with weight management behaviors in individuals attempting a self-guided weight loss effort.

To our knowledge, however, undermining attitudes and undermining behaviors have not been examined within the context of a behavioral weight loss (BWL) intervention, which is a first-line treatment for weight loss. It is unclear how partner undermining attitudes and behaviors may change over the intensive period (i.e., first 3 months) of treatment when individuals are rapidly adopting new behaviors, especially given that BWL programs do not typically devote much intervention time to psychoeducation on or skills development to address undermining. Further investigation is warranted to clarify these dynamics of social undermining from romantic partners, particularly among adults attempting weight loss through structured programs.

In the current study, all participants were required to nominate an adult to serve as their support partner throughout the intervention, and there was variability in whether partnered participants nominated their romantic partner or another person in their life. This provides a unique opportunity to examine whether the experience of undermining attitudes and undermining behaviors from romantic partners differed between those who did and did not choose their romantic partner to serve in this support role. It may also be important to understand the associations between relationship quality and undermining, as relationship satisfaction could impact the degree to which individuals perceive partner undermining attitudes and behaviors. Examining these differences has clinical utility, as increasingly more studies attempt to include romantic partners in BWL interventions (e.g., Gorin et al., 2020).

The present study describes participants’ experiences of their romantic partner’s undermining attitudes and undermining behaviors during the intensive period of a BWL intervention. Aim 1 was to (a) characterize the extent to which participants endorsed experiencing romantic partner undermining attitudes and behaviors in BWL, and (b) test the hypothesis that undermining attitudes and behaviors would increase from baseline to 3 months (end of the intensive phase of the BWL treatment), given this is the period in which participants are rapidly adopting new behaviors. Aim 2 was to examine whether endorsement of romantic partner undermining attitudes and behaviors at (a) baseline and (b) across time differed by support person nomination status (i.e., whether the participant chose the romantic partner as their study support person) and relationship quality. We hypothesized that (a) romantic partner undermining attitudes and behaviors would be significantly lower at baseline among those who nominated their romantic partner as their study support person and reported a higher relationship quality and (b) that the romantic partner serving as the support person and higher relationship quality would buffer changes in undermining over time.

## Methods

### Participants

Participants in the present sub-study were a cohort of adults with overweight/obesity enrolled in a randomized controlled trial of a BWL intervention (*N* = 128) (Miller et al., 2023). Detailed procedures for the parent study have been previously published (Miller et al., 2023) and it was pre-registered at clinicaltrials.gov (NCT05180448). Participants were recruited nationally via advertising on social media, digital media, radio stations, and primary care practices. Inclusion criteria for the parent intervention included: age 18–70, BMI = 27–50 kg/m^2^, ability to safely engage in physical activity (i.e., walk 2 city blocks without stopping), access to a smartphone and a stable internet connection, and completion of enrollment procedures. Participants were also required to nominate one adult friend or family member (age 18 +) to serve in a supporting role in the program; this person did not have to have any interest in their own weight loss and did not receive weight loss coaching. Participants were encouraged to nominate a support partner with whom they had a supportive, stable relationship. Exclusion criteria were as follows: any medical or psychiatric condition that could pose a risk to the participants, cause weight change, or limit the ability to comply with the program; the use of a medication that could cause significant weight change; a history of bariatric surgery; currently pregnant or breastfeeding or plans to become pregnant in the next 24 months; and weight loss ≥ 5% in the past 3 months. All study procedures were approved by the Institutional Review board at the supporting institution and all participants provided informed consent and were compensated for completing study assessments. All participants in this sub-study needed to report having a romantic partner, operationalized as a spouse, boyfriend/girlfriend, or significant other. Of all individuals who participated in the parent study during this data collection period, 128 (82.1%) reported having a romantic partner and were included in the sub-study.

### Procedures

The current study was a sub-study of an ongoing, randomized controlled trial of a BWL intervention (Miller et al., 2023). The 24-month parent trial includes a curriculum consisting of core BWL skills (e.g., goal setting, self-monitoring of eating and exercise) that is remotely delivered across group sessions (90 min via Zoom videoconferencing), coaching calls, and text messages. The curriculum was adapted from the Diabetes Prevention Program (Diabetes Prevention Program Research Group, 2002) and Look AHEAD (Wadden et al., 2006) protocols. Group sessions were led by Masters- or doctoral-level clinicians in psychology or a related field and occurred weekly for the first 12 weeks of the program, and once every 3 months thereafter. Participants were instructed to gradually increase their moderate-to-vigorous physical activity to reach a threshold of 250 min/week and encouraged to lose weight at a rate of 1–2 lbs per week to achieve a 10% weight loss. Participants also received monthly text messages from their coach and one phone call (15 min) from their study coach during the first 3 months of the program. After the initial 3-month period of intervention, participants were provided with either one group session or one brief phone call from their coach each month for the remaining 24 months.

All participants nominated one friend/family support partner in the program. Participants in the parent study were randomly assigned in 2 × 2 × 2 factorial design to have digital device data (food logs, activity minutes) shared (ON) or not shared (OFF) with their study coach, a subset of their group members, and their support partner. Nominated friend/family support partners participated in one remote webinar at baseline to learn strategies for effective social support, including providing validation, empathy, and tangible assistance. Beyond the three-month period assessed in this sub-study, support partners also received three additional webinars across the 24-month parent study on effective social support. Support partners also received monthly messages from the program that began at week 6, such that they received messages twice during the period of the sub-study. Those in support partner data sharing OFF received standardized education on key principles of the program and those in data sharing ON received the monthly data report. Of note, the content of the webinar and text messages did not provide psychoeducation about or address social undermining. The current study examined undermining attitudes and undermining behaviors during the intensive phase (i.e., first three months) of the program across all participants with a romantic partner.

### Measures

All data were collected via Research Electronic Data Capture (REDCap), a secure web-based software platform designed to support data capture for research studies approved for use by the supporting institution (Harris et al., 2009). Assessments for the current study were conducted at baseline and 3 months. All variables collected about romantic partners were reported by participants and romantic partners did not complete any assessment measures, which was consistent with parent study requirements.

*Participant and relationship characteristics*. All participants reported demographic characteristics at baseline. Participants also provided information on their romantic partner’s gender identity (male, female, non-binary/other), length of their romantic relationship (years), information pertaining to the quality of their romantic relationship, comfort discussing weight with their partner, and their romantic partner’s personal interest in losing weight at baseline using investigator-derived measures. Participants rated their relationship quality using one investigator-derived item on a 7-point Likert type scale from 0 (very negative) to 6 (very positive), with higher scores indicating higher relationship quality. Participants rated their comfort with discussing weight with their romantic partner using one investigator-derived item on a 7-point Likert type scale ranging from 0 (not at all comfortable) to 6 (completely comfortable), with higher scores indicating greater comfort. Participants indicated whether they believed their romantic partner was interested in losing weight themself as not at all interested, somewhat interested, or very interested, with an option to indicate not sure if the participant was not sure whether their romantic partner was interested in losing weight themself. Participants also indicated whether they nominated their romantic partner as their support person in the study (1 = yes, 0 = no).

*Partner undermining attitudes and behaviors*. Partner undermining attitudes and behaviors were self-reported by participants at baseline and 3 months. Only participants reported partner undermining attitudes and behaviors, and romantic partners did not report on their own undermining attitudes and behaviors. As such, this measures the participant’s *perception* of their romantic partner’s undermining attitudes and behaviors. To measure partner undermining attitudes, we used 33 statements developed by Dailey et al. (2022) on a Likert-type scale from 1 (strongly disagree) to 7 (strongly agree). Attitudes were categorized into 6 domains: no need for weight loss (4 items; α =.90; e.g., “My partner doesn’t think I need to lose weight”); imposition on partner (7 items; α =.96; e.g., “My partner feels my weight loss efforts create burdens for him/her”); relational fears (5 items; α =.94; e.g., “My partner worries that I will find him/her less attractive if I lose weight”); unhealthy practices (9 items; α =.93; e.g., “My partner worries that my weight loss pursuits will negatively affect my health”); negative comparison (4 items; α =.87; e.g., “My weight loss efforts will make my partner question his/her own diet and exercise behaviors); and undue stress (4 items; α =.86; e.g., “My partner feels my weight loss pursuits create a lot of stress for me”). Items in each domain were averaged, with higher scores indicating higher endorsement of undermining attitudes. A total partner undermining attitude score was also calculated as the average of the 6 domain scores (α =.71).

To evaluate partner undermining behaviors, participants rated two investigator-derived items measuring criticism/complaint of and behavioral interference of weight control on Likert-type scale from 1 (never) to 7 (very often): “My partner’s eating and exercise behaviors interfere with my weight loss efforts” and “My partner complains about my eating and exercise behaviors.” Criticism/complaint of weight control and behavioral interference of weight control have been identified by previous studies as two key factors of undermining behaviors (Dailey et al., 2022), and are represented in other measures of undermining behaviors (e.g., Sallis et al., 1987). Because behavioral undermining is a more established construct than undermining attitudes, this two-item measure assessing the core components of undermining behaviors was utilized to reduce participant burden instead of a more comprehensive measure (Dailey et al., 2022). Higher scores indicated perceptions of greater romantic partner interference and complaint of weight loss efforts. Interference and complaint were examined as separate domains and averaged to create a total undermining behavior score (α =.70), with higher scores indicating more frequent partner undermining behavior.

### Analysis plan

Data were analyzed in SPSS version 29.0. (IBM Corp., 2022). Analyses were run at a significance level of.05, all reported *P* values were based on two sided hypotheses, effect sizes were reported for all analyses, and Bonferroni corrections were used to control for multiple comparisons. Assumptions for parametric tests were assessed. Outliers with a standardized residual of ≥|3| were excluded in analyses for hypotheses tests, including one significant outlier for undermining attitudes and one for undermining behaviors. Undermining attitudes and behaviors showed deviations to normality upon visual inspection. As such, all analyses were replicated using parametric tests with log-transformed dependent variables and non-parametric alternatives (Friedman test, Mann–Whitney U test). Given that the pattern of results using log-transformed dependence variables and non-parametric alternatives did not differ from parametric results and that parametric methods can be robust to non-normality (Blanca et al., 2023), parametric analyses using non-transformed variables were retained to improve interpretability. Participant gender, age, and race were not significantly related to undermining attitudes or behaviors at baseline or 3 months (ps >.05), and thus were not included as covariates in analyses.

Descriptive statistics (means, SDs, frequencies) were calculated to summarize participant demographic and relationship characteristics, and endorsement of undermining attitudes and undermining behaviors at baseline and 3 months (Aim 1a). To examine changes in undermining attitudes and undermining behaviors from baseline to 3 months (Aim 1b), we ran repeated measures ANOVAs controlling for experimental conditions. Of note, aims incorporating the effect of experimental condition would have been underpowered and beyond the scope of the paper. The differences in intervention in experimental conditions in the first 3 months of the study were also very modest, so differences in outcomes were not yet expected. Independent samples t-tests were run to examine whether total undermining attitudes and total undermining behaviors reported at baseline differed by relationship quality and between those who did and did not choose their romantic partner as their support person in the BWL intervention (Aim 2a). Relationship quality was transformed to a dichotomous variable to create meaningful groupings of lower relationship quality and higher relationship quality. Lower relationship quality was operationalized as a score of 4 or less and higher relationship quality was operationalized as a score of 5 or higher on the 7-point Likert type scale from 0 (very negative) to 6 (very positive). In this sample of participants, relationship quality was generally high, with the majority of participants (n = 101; 78.9%) self-reporting relationship quality as a 5 or 6. As such, these groupings were created to separate those who reported less positive or mixed quality (*n* = 27, 21.1%) from those reporting a high positivity in their relationship (*n* = 101; 78.9%). We considered creating a tertile variable to separate those with the lowest relationship quality from those with satisfactory and high relationship quality, however only six participants (4.7%) reported their relationship quality as a score of 3 (i.e., mixed) or lower, and this grouping would have been too unequal to examine in comparison to the others.

To examine Aim 2b, interaction effects of support person nomination status (1 = romantic partner is designated support person; 0 = romantic partner is not designated support person) or relationship quality (1 = lower relationship quality; 2 = higher relationship quality) and time on undermining attitudes and behaviors were added to Aim 1b models to examine potential moderation effects.

## Results

### Participant characteristics

Baseline participant and romantic relationship characteristics are presented in Table 1. The sample was majority White, non-Hispanic females in long-term (*M* = 25.4 ± 13.1 years) heterosexual relationships. Perceived romantic relationship quality (*M* = 5.2 ± 1.0) and comfort speaking about weight with romantic partner (*M* = 4.7 ± 1.7), measured on a Likert-type scale from 0 (low) to 6 (high), were high among the sample.

**Table 1:** Baseline index participant and romantic relationship characteristics (N = 128)

*Index participant characteristics*	
Age (*M* ± *SD*)	54.4 ± 10.7
BMI (*M* ± *SD*)	34.2 ± 5.1
Sex, *n* (%)	
Male	35 (27.3%)
Female	93 (72.7%)
Gender identity, n (%)	
Male	34 (26.6%)
Female	92 (71.9%)
Trans woman	1 (0.8%)
Genderqueer	1 (0.8%)
Race, *n* (%)	
American Indian/Alaska Native	1 (0.8%)
Asian	4 (3.1%)
Black/African-American	12 (9.4%)
White	110 (85.9%)
Unknown/Prefer not to say	1 (0.8%)
Ethnicity,* n* (%)	
Hispanic	2 (1.6%)
Non-Hispanic	126 (98.4%)
Employment status, *n* (%)	
Full-time	87 (68.0%)
Part-time	11 (8.6%)
Occasional	7 (5.5%)
Disability/SSI	6 (4.7%)
No income	17 (13.3%)
Romantic relationship characteristics (as reported by the study participant only)*	
Romantic partner gender identity, *n* (%)	
Male	92 (71.9%)
Female	35 (27.3%)
Non-binary	1 (0.8%)
Romantic partner interest in weight loss for themself, *n* (%)	
Not at all	27 (21.1%)
Somewhat interested	61 (47.7%)
Very interested	39 (30.5%)
Not sure/not applicable	1 (0.8%)
Relationship length in years (*M* ± *SD*)	25.4 ± 13.1
Relationship quality (*M* ± *SD*)*	5.2 ± 1.0
Comfort talking about weight with romantic partner (*M* ± *SD*)	4.7 ± 1.7

^*^Romantic relationship characteristics were reported by study participants only as the participant’s perception of the relationship characteristics. Romantic partners did not complete any assessments for this study. Relationship quality was rated on a scale from 0 (Very Negative) to 6 (Very Positive); Comfort in talking with romantic partner about their weight was rated on a scale from 0 (Not at all) to 6 (Very Comfortable)

### Aim 1: Social undermining descriptives and change across time

Mean social undermining attitudes and behaviors at baseline and 3 months are presented in Table 2. Repeated measures ANOVAs controlling for experimental conditions showed a significant reduction in total undermining attitudes (*F*(1,113) = 15.8, *p* <.001, η^2^ =.12) and undermining behaviors (*F*(1,113) = 20.8, *p* <.001, η^2^ =.16) from baseline to 3 months across all participants. Analyses by sub-domain of undermining attitude with a Bonferroni correction showed that imposition on partner (*p* <.001), negative comparison (*p* =.005), and undue stress (*p* =.002) significantly decreased across time, while no need for weight loss, unhealthy practices, and relational fears did not significantly change (*p*s >.05). Analyses by sub-domain of undermining behavior with a Bonferroni correction showed that both criticism/complaint (*p* =.003) and interference behaviors (*p* <.001) significantly decreased from baseline to post-treatment.

**Table 2:** Mean change in undermining attitudes and behaviors from baseline to 3 months (N = 117)

	Baseline *M* (*SD)*	3 Months *M* (*SD)*	*M* change	η^2^
**Total Undermining Attitudes**	2.2 (0.7)	2.0 (0.5)	− 0.2*	.13
No Need for Weight Loss	3.2 (1.4)	3.3 (1.3)	+0.1	.02
Imposition on Partner	2.0 (1.2)	1.6 (1.0)	− 0.4*	.13
Relational Fears	1.3 (0.7)	1.2 (0.7)	− 0.1	.04
Unhealthy Practices	1.3 (0.6)	1.2 (0.4)	− 0.1	.04
Negative Comparison	2.6 (1.3)	2.3 (1.2)	− 0.3*	.07
Undue Stress	2.5 (1.2)	2.1 (1.0)	− 0.3*	.08
**Total Undermining Behaviors**	2.3 (1.2)	1.9 (1.1)	− 0.4*	.13
Criticism/complaint	1.8 (1.1)	1.5 (1.1)	− 0.3*	.08
Interference behaviors	2.9 (1.6)	2.3 (1.5)	− 0.6*	.11

^*^Significant at *p* <.05 based on a Bonferroni correction controlling for multiple comparisons

### Aim 2: Support partner nomination status and relationship quality comparisons

Of all included participants with a romantic partner, 60.2% (*n* = 77) chose to nominate their romantic partner as their study support person, while the remaining 39.8% (*n* = 51) chose another family member or friend for the role. Regarding relationship quality, 78.9% (*n* = 101) reported having a higher relationship quality (operationalized as a score of 5 or higher on a scale from 0 = very negative to 6 = very positive) and 21.1% (*n* = 27) reported having a lower relationship quality (score ≤ 4). Mean baseline partner undermining attitudes (*t*(125) = 0.63, *p* =.53, *d* =.11) and behaviors (*t*(126) = 1.88, *p* =.06, *d* =.34) did not significantly differ between those who did and did not choose their romantic partner as their support individual. Furthermore, mean baseline partner undermining attitudes (*t*(125) = 1.37, *p* =.17, *d* =.30) and behaviors (*t*(125) = 1.72, *p* =.05, *d* =.43) did not significantly differ between those with higher and lower relationship quality. When support person nomination status and relationship quality were added to Aim 1b ANOVAs, interactions between time and support person nomination status or relationship quality on undermining attitudes and undermining behaviors were not significant (*p*s >.05).

## Discussion

The present study examined social undermining by romantic partners during the intensive phase of a BWL intervention. Social undermining includes undermining attitudes, or a romantic partner’s negative beliefs about their significant other’s weight loss efforts, and undermining behaviors, which include overt complaints or interfering behaviors that are incompatible with weight control efforts (Dailey et al., 2022; Mackert et al., 2011). There is some evidence to suggest that romantic partner undermining is a critical barrier to weight loss (Harp, 2013; Theiss et al., 2016), but there has been little investigation of romantic partner undermining among individuals enrolled in a BWL intervention, which is a first-line treatment for overweight and obesity (Wadden et al., 2020). BWL interventions do not typically provide much psycho-education or skills management targeted toward social undermining. This study was the first of the authors’ knowledge to examine and quantify changes in romantic partner undermining during the intensive phase of a BWL intervention.

At baseline and 3 months, participants generally disagreed that their romantic partner had undermining attitudes about their weight control attempts and reported low frequency of undermining behaviors. Dailey et al. (2022) reported similarly low endorsement of undermining attitudes and behaviors (means between 2 and 3 on a scale from 1 (low) to 7 (high)) in their study of individuals undergoing a self-guided weight loss attempt. Despite qualitative studies highlighting the detrimental nature of social undermining for weight control, studies that have quantified social undermining during weight loss have found that these events are infrequent (Dailey et al., 2022; Harp, 2013; Metzgar et al., 2015; Theiss et al., 2016). This may suggest that participants believe that their romantic partner holds relatively neutral or positive beliefs about their weight control efforts and engages in little behavioral interference or criticism. Participants may also be hesitant to report romantic partner undermining attitudes and behaviors because of social desirability or may particularly highlight recent or upsetting instances in qualitative studies. Future studies should use mixed methods approaches to elucidate the discrepancy between qualitative and quantitative findings. Alternatively, there could be a need to improve the measurement of social undermining by refining the item pool or assessing undermining events more frequently using methods such as daily ambulatory assessment (Roordink et al., 2023).

Despite the perceptions of minimal partner undermining reported, results showed that there was a significant reduction in total undermining attitudes and behaviors at 3 months compared to baseline with medium effect sizes. It is promising that participants did not perceive an increase in partner undermining attitudes or behaviors during a period of BWL in which they rapidly change eating and exercise behaviors in a way that may be misaligned with current norms in their social environment (Deslippe et al., 2023). In contrast, a study by Wang et al. (2014) found no significant changes in social undermining from any source (friends, family, coworkers) across a 2-year weight gain prevention intervention. There was no explicit content about social undermining presented in this intervention; however, it could be that the group-based nature of the intervention with frequent contacts and support buffered against partner undermining. Given that participants reported on their perception of undermining from their romantic partner, rather than the romantic partner reporting on their own behaviors, it is unclear whether romantic partner’s undermining attitudes and behaviors *actually* declined throughout the intervention or whether participants *perceived* a decline. Previous research has shown that perceptions of support, rather than receipt of support or attempts to quantify support resources, are much stronger predictors of health behavior engagement (Uchino et al., 2018). Therefore, the perception of a decline may be quite beneficial for weight control. It is promising that BWL interventions that employ high levels of support may improve social undermining without explicit content designed to do so. Perhaps as participants make weight control changes in the context of a structured, supportive environment, they enhance their self-efficacy and mastery of skills such that the perception of undermining becomes muted, even if it still exists to the same degree. Importantly, although these decreases in undermining were statistically significant, they were small, and the clinical significance of these findings cannot be quantified. Future research should replicate these findings in BWL interventions with varying levels of support, compare results to social undermining experienced in other weight control programs (e.g., self-guided weight loss, commercial weight loss), and measure clinical significance of findings by examining relationships to weight control behaviors.

Surprisingly, partner undermining attitudes and behaviors did not differ by the participant’s decision to nominate their romantic partner as their study-designated support person or relationship quality and nomination status and relationship quality did not moderate change in undermining over time. Participants who nominated their romantic partner as their study support person or reported higher relationship quality did not report significantly lower partner undermining at baseline than those who chose another individual in their life or reported lower relationship quality as hypothesized. Furthermore, reductions in undermining across time did not depend on whether the romantic partner was engaged as the support person in the program or on baseline relationship quality. Therefore, it is promising that romantic partner undermining can decrease in BWL even without the romantic partner serving in a designated support role and receiving education about effective support. It could be that participants focused on traits other than undermining when nominating a study support individual, such as frequency of contact, convenience, or shared goals, which is supported by previous research from our team that those who chose romantic partners as their support person, compared to those who chose other family members, reported significantly higher frequency of communication (Crane et al., 2024). Participants may have also retained a biased focus on positive social support *alone* when making their support role decision*.* It is also plausible that individuals avoided nominating someone they perceived as undermining altogether, such that those with minimally undermining partners nominated them for the role and those with undermining romantic partners chose a different individual. Finally, as support partners had to consent for participation, it could be that undermining romantic partners declined to participate, requiring participants to choose an alternative. It is also promising that participants perceived a reduction in undermining across time, regardless of initial relationship quality. However, there was little variability in reported relationship quality in this sample, with approximately 80% of participants reporting highly satisfactory relationships, and future research should examine undermining in lower quality relationships.

The present study has several notable strengths, including investigation of partner undermining within a BWL intervention and examination of partner undermining attitudes in conjunction with behaviors. There are also several limitations to the present study. First, the sample consisted of primarily White women in midlife and older adulthood and the romantic relationships represented in the sample were largely high-quality, long-standing, heterosexual partnerships. More research on the dynamics of social undermining in weight loss in the context of queer relationships is needed, such as that represented by Novak et al. (2021), and social undermining across relational stages or in differing cultural contexts (e.g., collectivist cultures) should be examined in future studies. Furthermore, this study utilized an investigator-derived measure of undermining behaviors to reduce participant burden, and these results should be replicated with more comprehensive measurement of both undermining attitudes and behaviors. Additionally, this study focused on measuring the frequency of social undermining in romantic partnerships, but it is certainly plausible that romantic partners engage in both support and undermining. This study also did not examine the relationship between romantic partner undermining and weight loss outcomes (e.g., weight loss, self-monitoring behaviors). Future studies should examine both supportive and undermining romantic partner attitudes and behaviors, the participant’s reaction to supportive and undermining behaviors, and the relationship to weight loss outcomes to better understand the role of social undermining in weight loss. Finally, although undermining from a romantic partner may be particularly salient given the interdependent nature of the relationship, it is possible that participants in the present study experienced social undermining from other parties in their social network, such as friends or coworkers. Next steps in this line of research could work to conceptualize social undermining in BWL from other important people in the participant’s social network.

The present study adds to a growing body of literature examining social undermining as a negative social influence that is distinct from a lack of positive social support or negative social control and has the potential to disrupt engagement in behaviors for weight loss. The present study found that undermining of weight loss efforts by a romantic partner serving in a formal support role was relatively infrequent at the beginning of and during a BWL intervention. Participants reported a significant reduction in romantic partner undermining during the initial phase of a BWL intervention, highlighting the potential of group-based BWL interventions to mitigate negative social influences even without explicit content focused on addressing social undermining. Future research should examine romantic partner undermining across time in varying weight control and relationship conditions to uncover potential context-dependent differences and capture undermining at the daily or weekly level to reduce recall bias and enhance ecological validity.

## Data Availability

Access to data available upon reasonable request.
